# Moyamoya Disease and Flow Velocity

**DOI:** 10.31662/jmaj.2022-0191

**Published:** 2022-12-26

**Authors:** Teiji Tominaga

**Affiliations:** 1 Department of Neurosurgery, Tohoku University Graduate School of Medicine, Sendai, Japan

**Keywords:** Moyamoya disease, Moyamoya syndrome, Moyamoya vasculopathy, Flow velocity

Moyamoya disease (MMD) and moyamoya syndrome (MMS) remain a great challenge for both scientists and clinicians. Although the identification of *RNF213*, the susceptibility gene for the onset of MMD, was a breakthrough, the exact mechanisms of the initiation and worsening of moyamoya vasculopathy remain unknown. Multiple factors, including genetic or environmental factors such as autoimmune mechanisms, chronic inflammation, infection, and/or irradiation, are believed to be required for MMD development. This finding is supported by experiments that demonstrated that mice with the genetic deletion of *Rnf213* or those carrying human *RNF213* c.14576G>A knock-in did not show any abnormal cerebrovascular structures ^(1), (2)^.

In general, MMD pathophysiology is understood as a primary narrowing of the carotid fork and secondary development of collateral circulation as compensation for the decreased cerebral blood flow. In this issue of *Japan Medical Association Journal*, Dr. Abumiya and his colleagues reported a possible trigger effect of increased flow velocity for moyamoya vasculopathy^(3)^. The authors reviewed diseases complicated by MMS and found increased flow velocity without stenotic changes. They also described increased flow velocity of the middle cerebral artery in females and children, who predominantly suffer from MMD. The authors also demonstrated a greater flow velocity in the anterior circulation compared with posterior circulation. They hypothesized that increased flow velocity triggers the development of moyamoya vasculopathy. Because flow velocity changes following stenotic change, this unusual hypothesis is intriguing.

However, it may be difficult to establish flow velocity as the primary change. Many factors, including viscosity of blood, diameter of blood vessels, and demand of blood flow, may affect flow velocity. For example, the authors described the difference in the velocity in the anterior circulation and posterior circulation. However, the diameter of the anterior circulation and posterior circulation is different, and flow velocity should be greater in the anterior circulation. Moreover, flow velocity should be greater outside the vascular curve, but vascular change in MMD is not outside-specific. In contrast, changes in flow velocity modify shear stress and other factors, resulting in injury or dysfunction of vascular endothelial cells. The change in flow velocity might be just an intermediate step. The lack of animal models prevents the accumulation of robust evidence and the elucidation of various pathophysiologic mechanisms.

However, the authors’ hypothesis gives us an opportunity for discussion. I look forward to future research developments that will elucidate the cause of MMD.

## Article Information

### Conflicts of Interest

None
